# Lymph node metastases in early gastric cancer: Japanese Gastric Cancer Treatment Guidelines can be used for endoscopic resection in the West

**DOI:** 10.1055/a-2749-4324

**Published:** 2025-12-04

**Authors:** Edward Young, Louisa Edwards, Aashish Maurya, Andrew Ruszkiewicz, Hamish Philpott, Rajvinder Singh

**Affiliations:** 1 1066 School of Medicine, Faculty of Health and Medical Sciences, University of Adelaide Press, Adelaide, Australia; 2 3187 Department of Gastroenterology, Lyell McEwin Hospital, Elizabeth Vale, Australia; 3 1062 Department of Gastroenterology, Royal Adelaide Hospital, Adelaide, Australia; 4 3187 Tissue Pathology, Lyell McEwin Hospital, Elizabeth Vale, Australia; 5 3187 Gastroenterology and Hepatology, Lyell McEwin Hospital, Elizabeth Vale, Australia

**Keywords:** Endoscopy Upper GI Tract, Precancerous conditions & cancerous lesions (displasia and cancer) stomach, Endoscopic resection (ESD, EMRc, ...), GI Pathology

## Abstract

**Background and study aims:**

The eCura system has been shown to accurately delineate early gastric cancers with negligible risk of lymph node metastases, which, therefore, would be considered endoscopically cured. However, this classification was based predominantly on data from high-incidence Eastern countries. We sought to assess whether these criteria can be safely applied in a Western population.

**Patients and methods:**

Data were retrospectively recorded for patients who underwent gastrectomy in four Australian tertiary centee over two decades. Demographic data, lesion characteristics (size, differentiation, invasion depth, lymphovascular invasion, and ulceration) as well as number of lymph node metastases was recorded. Patients given neoadjuvant chemotherapy were excluded.

**Results:**

A total of 1,465 gastrectomy specimens were reviewed, including in 558 patients who underwent resection of gastric adenocarcinoma without neoadjuvant chemotherapy (mean age 67.9, 64.2% male). Of these, 101 (18.1%, confidence interval [CI] 15.4%-21.9%) had T1 disease (T1a = 30, T1b = 71). Of the lesions, 11.5% (n = 64, CI 9.1%-14.4%) met the 2021 Japanese "absolute criteria" for endoscopic resection, with 7.8% of these (n = 5, CI 3.4%-17%) having positive lymph nodes at gastrectomy. Of them, 9.9% (n = 55, CI 7.6%-12.6%) would have been considered eCura A or B, with none of these having positive lymph nodes at gastrectomy.

**Conclusions:**

The eCura system for defining endoscopic curability could have been safely applied in this Western population. Even in Western countries, patients with early gastric cancer that meets Japanese guidelines for endoscopic resection should, where possible, undergo en bloc endoscopic submucosal dissection. Lesions classified histologically as eCuraA or B should be considered endoscopically cured.

## Introduction

Gastric cancer remains a significant global health concern, ranking as the fifth most common cancer and the fourth leading cause of cancer-related mortality worldwide 1 . In high-prevalence Eastern countries such as Japan and Korea, nationwide gastric cancer screening programs have reduced the risk of gastric cancer mortality by up to 40% 2 . This is in large part due to early detection of gastric cancer and premalignant gastric lesions, offering a pivotal opportunity for curative endoscopic treatment and vastly improving survival. With the advent of endoscopic resection techniques such as endoscopic mucosal resection (EMR) and endoscopic submucosal dissection (ESD), concerns have arisen regarding the potential for residual lymph node metastases (LNM) that would have been incorporated into a gastrectomy specimen but would not be resected during endoscopic treatment. The safety of these endoscopic resection techniques therefore hinges on the accurate prediction of the likelihood of LNM based on characteristics of the primary lesion itself.

Early gastric cancer (EGC) includes adenocarcinoma confined to the mucosa or submucosa. In the most recent Japanese Gastric Cancer Treatment Guidelines (6 ^th^ Edition), absolute indications for ESD have been widened to include lesions that would previously have been considered “expanded criteria” lesions ( Table 1 ) 3 . However, the overall rate of LNM in T1 gastric cancers remains unacceptably high at more than 20%, with LNM as high as 8% in those meeting the absolute indication for ESD described above 4 5 . Therefore, EGCs must be further stratified after resection according to their risk of LNM to guide appropriate management. Because of widespread gastric cancer screening in Eastern countries, there is now a wealth of data on EGCs and prediction of LNM. This was the basis for development of the eCura system in Japan in 2017, stratifying lesions according to tumor size, invasion depth, differentiation, lymphovascular invasion, ulceration, and horizontal and vertical margin clearance 6 . In the most recent edition of the Japanese Cancer Treatment Guidelines, lesions are classified according to the eCura system as eCuraA, eCuraB, eCuraC-1 and eCuraC-2 ( Table 2 ) 3 .

**Table:** **Table 1** Indications for endoscopic resection according to the 6th Edition of the Japanese Gastric Cancer Treatment Guidelines 3 .

Indication	Definition
Absolute indication for EMR/ESD	Differentiated-type adenocarcinoma without ulcerative findings, clinically T1a, diameter ≤ 2 cm
Absolute indication for ESD	Differentiated-type adenocarcinoma without ulcerative findings, clinically T1a, diameter > 2 cm
	Differentiated-type adenocarcinoma with ulcerative findings, clinically T1a, diameter ≤ 3 cm
	Undifferentiated-type adenocarcinoma without ulcerative findings, clinically T1a, diameter ≤ 2 cm
Expanded indication for ESD	Locally recurred lesion, clinically T1a, differentiated-type
Relative indication for ESD	Tumors that do not fulfill the absolute or expanded indications in those who have high operative risk
EMR, endoscopic mucosal resection; endoscopic submucosal dissection.	

**Table:** **Table 2** The eCura system as described in the 6th Edition of the Japanese Gastric Cancer Treatment Guidelines 3 .

eCura classification	Definition	
eCuraA	No ulceration	En bloc resection Any tumor size if differentiated-type dominant ≤ 2 cm if undifferentiated-type dominant pT1a (intramucosal) Clear horizontal and vertical margins No lymphovascular infiltration
	Ulceration	En bloc resection Differentiated-type dominant pT1a (intramucosal) Tumor size ≤ 3 cm Negative horizontal and vertical margins No lymphovascular invasion
eCuraB		pT1b (SM1): < 500 µm from the muscularis mucosa Negative horizontal and vertical margins Tumor size ≤ 3 cm Differentiated-type dominant No lymphovascular invasion
eCuraC-1		Meeting eCuraA or B, histologically differentiated type Either not resected en bloc or positive horizontal margin
eCuraC-2		Not meeting the criteria above

Based on data from more than 5000 gastrectomy specimens in Japan, risk of LNM in eCuraA gastric cancers was 0% with a 95% confidence interval (CI) of 0% to 0.7% 7 . In eCuraB cancers, the risk of LNM was also 0%, although the 95% CI was marginally wider at 0% to 2.6% 7 . As a result, the Japanese Gastric Cancer Treatment Guidelines consider eCuraA and B histology to be curatively resected and to not require further treatment. For eCuraC-1 lesions, lymph node risk remains extremely low; however, pathological analysis is less conclusive in piecemeal resections, whereas lesions with positive horizontal margins are at risk of residual disease and local recurrence. The Japanese guidelines, therefore, suggest considering either repeating an ESD with wider margins, surgical resection, or close observation in these patients, depending on patient and lesion characteristics. In eCuraC-2 lesions, risk of LNM increases, so suitable patients should be considered for surgical resection. Nevertheless, in many patients, LNM risk remains less than 10% and an estimate can be predicted based on the original eCura system because the risk may still outweigh the benefits of surgical resection in high-risk surgical candidates 6 .

Despite the exhaustive data from Eastern populations, limited evidence exists on the applicability of these guidelines in Western populations. Historically, there has been concern that gastric cancer in the Western population may have differing tumor biology and a more aggressive phenotype, with endoscopic treatments, therefore, less likely to be curative 8 . This is, in part, extrapolated from the stark differences in gastric cancer survival between the East and the West, with 5-year survival in the West ranging from 10% to 30% compared with as high as 77% in Korea 9 10 11 . However, advances in diagnosis and treatment of EGC in the East may also influence this survival difference. To address these concerns, we sought to establish whether the Japanese Gastric Cancer Treatment Guidelines and the eCura system could be safely applied in a Western population based on local gastrectomy data.

## Materials and methods

This retrospective study included all patients who underwent gastrectomy for gastric cancer at one of four South Australian tertiary centers between 2000 and 2021. The statewide public hospital pathology database was used to review histopathology reports for all gastrectomy specimens during this period. Those with adenocarcinoma of any stage were included in the final analysis. Patients with gastroesophageal junction (GEJ) cancer or who received neoadjuvant chemotherapy prior to resection were excluded. Baseline data, including age at diagnosis, sex, and *Helicobacter pylori* status, as well as lesion characteristics including size, location, morphology, T stage, presence of ulceration, differentiation, lymphovascular invasion and perineural invasion were recorded. These datapoints were used to classify lesions histologically according to the eCura system 3 . Presence or absence, as well as number of LNM, was also documented. Histopathology was reported according to the Vienna classification system.

Data were analyzed using Stata version 18, with descriptive statistics for rates of LNM. A logistic regression model was used to calculate odds ratios (ORs) for LNM according to eCura classification, ulceration, differentiation, lymphovascular invasion, and lesion size. Similar logistic regression models were developed restricted to T1a or T1b lesions. This study was reviewed and approved by the Central Adelaide Local Health Network Human Research Ethics Committee (Reference Number: 15542). A waiver of consent was obtained given the nature of the study.

## Results

A total of 1,465 patients were included in the study with 812 patients excluded due to either non-malignant indications for gastrectomy, GEJ adenocarcinoma or treatment with neoadjuvant chemotherapy prior to surgery. 653 patients were then included in the final analysis (64.2% male), with a mean age of 67.9 ± 12.3. 558 patients had adenocarcinoma, with 58.2% (n = 325) having evidence of LNM on histopathological analysis ( Table 3 ). The majority of patients had advanced disease beyond the remits of endoscopic resection, with 72.1% (n = 402) having either T3 or 4 disease. Only 18.1% of patients (n = 101) had T1 disease, with 5.4% (n = 30) T1a and 12.7% (n = 71) T1b. Only two patients had multifocal T1 adenocarcinoma. Of the patients, 41.2% (230) had evidence of current or prior *H. pylori* infection on histopathology or serology, although many patients previously would have been treated elsewhere before their gastric cancer diagnosis.

**Table:** **Table 3** Baseline data for adenocarcinoma cases.

Characteristic	Level	Total n = 558
Age (years)		67.9 ± 12.3
Sex	F	200 (35.8)
	M	358 (64.2)
Cancer type	Differentiated	356 (63.8)
	Undifferentiated	202 (36.2)
T stage	1a	30 (5.4)
	1b	71 (12.7)
	2	55 (9.9)
	3	246 (44.1)
	4	156 (28.0)
*Helicobacter* status	Positive	230 (41.2)
	Negative	328 (58.8)

**Fig. 1:**
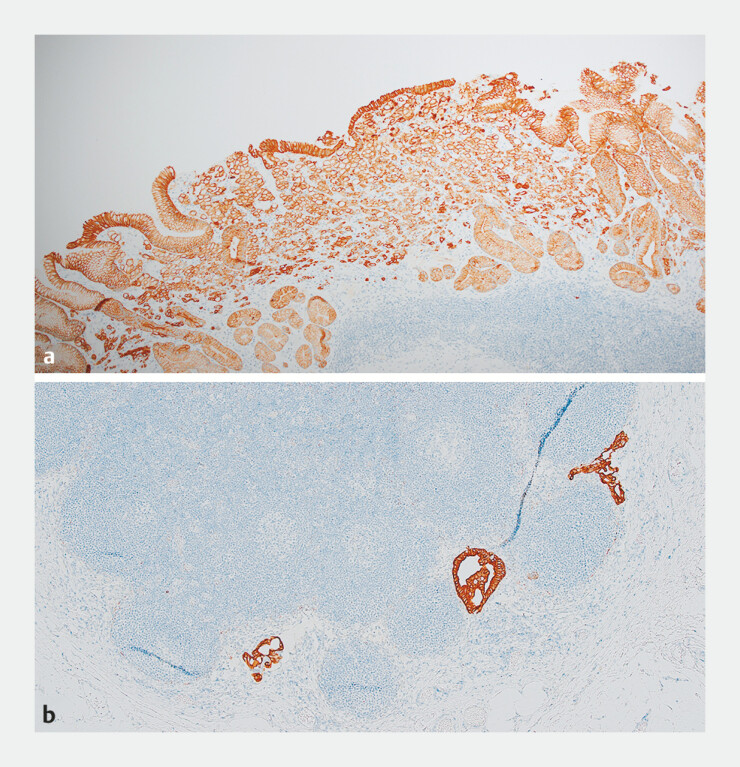
Histopathology from a patient with pT1a adenocarcinoma measuring 60 mm in maximal diameter. **a** AE1/3 immunostaining highlights invasive, often singly dispersed epithelial cells (poorly differentiated signet ring cell adenocarcinoma). Therefore, eCura C2 (diameter > 2 cm). **b** AE1/3 immunostaining demonstrating foci of metastatic adenocarcinoma in a regional lymph node.

Of the T1a adenocarcinomas, 20% (n = 6/30) had LNM ( Fig. 1 ), with similar rates seen in the T1b lesions at 19.7% (n = 14/71) ( Table 4 ). Forty-seven T1 lesions were ulcerated, with 38.3% (n = 18/47) having LNM compared with only 3.7% of lesions (n = 2/54) without ulceration. Of poorly differentiated T1 lesions, 27.8% (10/36) had LNM compared with 15.4% (n = 10/65) of well or moderately differentiated T1 adenocarcinomas. Lymphovascular invasion (LVI) was strongly correlated with LNM, with 68.2% (n = 15/22) of T1 lesions with LVI having LNM compared with 6.3% (n = 5/79) in the absence of LVI.

**Table:** **Table 4** Rate of lymph node metastases according to primary lesion characteristics.

Characteristic	Level	Number (%) with lymph node metastases
T stage	1a	6/30 (20.0)
	1b	14/71 (19.7)
	2	24/55 (43.6)
	3	161/246 (65.4)
	4	120/156 (76.9)
eCura (T1a or b cases)	A	0/22 (0.0)
	B	0/33 (0.0)
	C2	20/46 (43.5)
Size largest dimension (mm)	0–20	27/98 (27.6)
	21–40	90/161 (55.9)
	41–60	94/139 (67.6)
	61–80	51/69 (73.9)
	81+	62/89 (69.7)
Location (T1a or b cases)	Cardia	1/11 (9.1)
	Fundus	4/7 (57.1)
	Body	9/35 (25.7)
	Antrum	6/48 (12.5)
Macroscopic type	Elevated	5/33 (15.2)
	Depressed	12/52 (23.1)
	Polypoid	3/16 (18.8)

When stratified according to the eCura system described above, 9.9% (n = 55) of gastrectomy specimens contained eCura A or B adenocarcinoma. Most importantly, none of these had LNM on histopathologic analysis. Of the lesions, 8.2% (n = 46) were classified as eCura C2, with 43.5% (n = 20) of these patients having LNM ( *P* < 0.001).

The effect of lesion characteristics on the rate of LNM was compared using logistic regression, with poor differentiation (OR 2.57, *P* < 0.001), increasing size (OR as high as 7.45 for > 60-mm lesions, *P* < b0.001), lymphovascular invasion (OR 15.08, *P* < 0.001), and ulceration (OR 17.03, *P* < 0.001), all associated with increased prevalence of LNM. When the analysis was restricted to the T1 group only, characteristics associated with LNM included lymphovascular invasion (OR 31.71, *P* < 0.001) and ulceration (OR 17.03, *P* < 0.001), with poor differentiation (OR 2.11, *P* = 0.14) and lesion size ( *P* = 0.07) not reaching statistical significance ( Table 5 ).

**Table:** **Table 5** Logistic regression of characteristics associated with lymph node metastases in the T1 group.

Characteristic	Level	Number (%) with lymph node metastases	Odds ratio (95% confidence interval); *P* value
T stage	T1a	6/30 (20)	
	T1b	14/71 (19.7)	0.98 (0.34, 2.86); *P* = 0.97
Poorly differentiated	N	10/65 (15.4)	
	Y	10/36 (27.8)	2.11 (0.78, 5.71); *P* = 0.14
Lymphovascular invasion	N	5/79 (6.3)	
	Y	15/22 (68.2)	31.71 (8.86, 113.47); *P* < 0.001
Ulcerated	N	2/54 (3.7)	
	Y	18/47 (38.3)	16.14 (3.49, 74.52); *P* < 0.001
Size (greatest dimension mm)	0–20	5/50 (10.0)	Global P = 0.07
	21–40	7/27 (25.9)	3.15 (0.89, 11.14); *P* = 0.08
	41–60	6/16 (37.5)	5.40 (1.37, 21.26); *P* = 0.02
	61+	2/8 (25.0)	3.00 (0.47, 19.04); *P* = 0.24

## Discussion

This study highlights the safety of applying the Japanese Gastric Cancer Treatment Guidelines in a Western population. Although the rate of LNM according to the absolute indication for ESD remains unacceptably high, lesions should be further stratified according to the eCura system once histological evaluation of the resected specimen has been performed. This study demonstrates that the eCura system can accurately predict likelihood of LNM, even in the West, thereby identifying lesions that can be considered endoscopically cured. Despite concerns regarding phenotypic differences in gastric cancer between Eastern and Western populations, of the 55 patients with eCuraA or B gastric cancers, none had positive lymph nodes histologically. This, therefore, corresponds to 55 patients who could have theoretically avoided surgical gastrectomy.

Our data build upon the limited yet growing body of evidence regarding treatment of EGCs in Western populations. Feasibility of ESD previously has been demonstrated in an Australian cohort by Tate et al in 2019, who performed ESD for 135 adenomas and EGCs with en bloc and R0 resection rates of 94.8% and 86.7%, respectively 12 . Similarly, a German cohort in 2017 of 191 early gastric cancers had en bloc and R0 resection rates of 89% and 73.6%, respectively, even for expanded criteria lesions, although one patient developed LNM after ESD of a submucosal invasive expanded criteria lesion 13 .

With regard to prediction of LNM, three key Western studies have been recently published. A 2019 retrospective study from the United States examined 176 patients who underwent surgical resection for T1 gastric adenocarcinoma, with an overall LNM rate of 20.5% 14 . There were no patients with LNM who had met the standard criteria according to the latest Japanese Gastric Cancer Treatment Guidelines at that time, whereas 7.5% of expanded criteria lesions had positive lymph nodes. In the same year, Milhomem et al reported LNM in 13.48% of 178 Brazilian patients with T1 gastric cancer and emphasized risks of applying Eastern guidelines in a Western population, although this study also relied on the standard and expanded criteria rather than the eCura system 15 . Finally, in 2024, Morais et al published the first Western study assessing reliability of the eCura system for stratification of risk of LNM in non-curative ESDs, demonstrating excellent correlation (area under the receiver operating curve 0.90), superior to that of the original Japanese validation cohort 16 . Although this study did not stratify patients according to the A-C system described above, the findings mirrored our study, with no patients in the low-risk group having LNM compared with 53.1% of those in the high-risk group. To our knowledge, our study is the first in a Western population to stratify LNM risk according to the latest Japanese Gastric Cancer Treatment Guidelines eCura system.

Importantly, implementation of endoscopic resection techniques such as ESD for EGC relies on accurate endoscopic assessment and histology prediction. Accuracy of histology prediction using advanced mucosal imaging has been comprehensively demonstrated in the East; however, evidence remains limited in the West. Our 2025 study, including 232 gastric lesions assessed with high-magnification narrow-band imaging, demonstrated accuracy as high as 97% for identification of neoplastic lesions appropriate for endoscopic resection 17 . However, further studies are required to support accuracy of Western endoscopists as use of ESD becomes more widespread in the West.

It is crucial to underscore the significant morbidity and mortality associated with gastrectomy—risks that could potentially have been avoided for 55 patients in our cohort. A 2014 US study encompassing 2,580 gastrectomy patients from a large population database reported serious morbidity in 23.6% of patients, alongside a 30-day mortality of 4.1% 18 . Similarly, a 2013 study in England found 30-day mortality of 5.9% in 5,088 patients 19 . The largest Australian study in 2022 reported a comparatively favorable perioperative mortality of 2.1%, although it included only in-hospital mortality during the index admission 20 . Beyond mortality, the morbidity linked to gastrectomy often has a lasting impact on patient quality of life (QoL). A 2016 study from Karanicolas et al found that 55% of patients suffered significant impairment in global QoL post-surgery, with 20% to 35% continuing to report markedly diminished QoL up to 18 months later 21 . Although such outcomes can be challenging to quantify, they reflect the profound and long-term effects of gastrectomy, reinforcing the importance of finding safer, less invasive alternatives wherever possible.

There have been historical concerns that gastric cancers in Western populations behave differently than those in high-incidence Eastern countries, raising questions around the appropriateness of applying Eastern guidelines more broadly. As previously discussed, there are stark differences in the overall prognosis of gastric cancer, with 5-year survival in Western countries ranging from 10% to 30% compared with as high as 77% according to recent Korean data 9 10 11 . In addition, Asian migrants to the United States seem to have more localized disease and higher 5-year survival than White Americans, although these data should be interpreted with some caution because Asian migrants may be more likely to undergo surveillance endoscopy even in the United States 22 . Nevertheless, even in those with R0 resections in a Korean compared with United States population, the 5-year disease-specific survival was higher in the Korean population with a hazard ratio of 1.3 ( *P* = 0.008) based on multivariate analysis 23 . Further to this, immunohistochemical studies comparing Japanese and European gastric cancers have highlighted significant differences in pathologic staining, with a higher-risk staining profile in the European population 24 .

Some of these differences were reflected in our population, with overrepresentation of advanced-stage gastric cancers at diagnosis. Of the patients, 72.1% had at least T3 disease at diagnosis, with LNM present in 43.6% of T2 and 65.4% of T3 cancers. In comparison, systematic review data from Eastern populations have demonstrated rates of LNM as low as 21.9% and 41.9% for T2 and T3 gastric cancers, respectively 25 . Twenty percent of patients with T1 gastric cancer in our cohort overall had LNM, whereas recent Japanese data suggested this to be as low as 10.9% in the Japanese population 26 . Importantly, in the eCuraC2 group in our cohort, 43.5% of patients (n = 20/46) had LNM. Comparatively, in the initial validation dataset for the eCura score in a Japanese population, those in the eCuraC2 group had a risk of LNM between 4.9% and 27.3%, depending on individual lesion characteristics 6 . As such, although eCuraA and B lesions can be considered endoscopically cured, an additional degree of caution needs to be exercised with lesions outside these criteria in the West. In addition, there have been concerns regarding safety of endoscopic resection of gastric cancers due to the frequency of metachronous dysplasia and adenocarcinoma in the stomach, and therefore, increased risk of recurrence. In our study, only 2% of T1 gastric cancers were multifocal, reflecting a lower frequency in the Western population. Irrespective of that, there is now evidence for the safety of endoscopic resection in these patients, with no increase in LNM compared with solitary lesions, and no difference in overall survival (OS), with a significantly lower complication rate compared with gastrectomy 27 28 .

Although these differences may reflect biological differences in tumor types, multiple other factors likely contribute to the disparity in gastric cancer prognosis. Firstly, there are key differences in the reporting of histopathology, with the Japanese Gastric Cancer Association classification system using the term intramucosal adenocarcinoma for lesions that may be considered non-malignant adenomas according to the WHO or Vienna classification systems. This would inherently favor a worse prognosis for ‘early’ gastric cancers in the West 17 29 . Historical differences in sectioning of gastrectomy specimens may lead to under-sampling of the primary tumor, thereby underestimating the T stage, which would be reflected by an apparent increase in LNM for early-stage lesions. Similarly, there are likely to be differences in sectioning of gastrectomy specimens versus endoscopic resections, as precise characterization of the primary tumor risk profile (including lymphovascular invasion) is imperative when local lymph nodes have not been sampled. Another key factor has been the emergence of nationwide gastric cancer screening programs in the East, which have dramatically reduced gastric cancer incidence and improved survival. Recent Japanese data has shown a 22% reduction in gastric cancer incidence and 40% to 61% reduction in mortality as a result of nationwide endoscopic screening 2 30 . Finally, treatment-related factors influence OS survival data, given the wealth of experience treating gastric cancer in high-incidence countries. This is supported by a 1996 study comparing gastric cancer survival in Japanese migrants to the United States with a local population in Tokyo 31 . The significantly improved survival in the Tokyo group despite comparatively more advanced-stage disease suggests that treatment-related factors influence survival more than differences in tumor biology. Although there may be biologic differences between gastric cancers in the East and the West, they seem insufficient to preclude the Western population from minimally invasive endoscopic resection strategies according to Eastern guidelines.

This is the first study in a Western cohort to evaluate safety of the eCura system in determining endoscopic cure for early gastric cancers. We included a large cohort of surgically resected adenocarcinomas, ensuring the availability of lymph nodes in the resection specimen to carefully assess for even microscopic lymph node involvement. This study, therefore, should provide reassurance to interventional endoscopists undertaking endoscopic resections for these patients and supports use of the eCura system when considering the need for further therapy. Our study, however, does have limitations. Despite being a large cohort, because this was a Western population, most cancers were beyond the remits of endoscopic resection; therefore, the sample size for relevant endoscopically resectable cancers was small. In addition, we were not able to reliably assess which lesions met absolute indication for ESD according to the Japanese Gastric Cancer Treatment Guidelines, because endoscopic assessments were not available. 3 A further limitation is the differences in histopathological processing between endoscopic and surgical specimens, as well as between Eastern and Western countries. For example, in Western gastrectomy specimens, depth of submucosal invasion is not reported because lymph nodes also are resected regardless of that at time of gastrectomy. Nevertheless, this would predispose to underestimation of high-risk features in the primary tumor and in the absence of LNM in any eCura A or B lesions, this has, therefore, not impacted the key safety message of this study.

## Conclusions

Overall, our study illustrates the safety of implementing the Japanese Gastric Cancer Treatment Guidelines within a Western cohort, particularly highlighting the utility of the eCura system. With meticulous patient selection and appropriate endoscopic expertise, surgical resections with high morbidity and mortality could be avoided for eCuraA and B gastric cancers in the Western population.
